# Supplementary material for: A PCR amplicon-based SARS-CoV-2 replicon for antiviral evaluation
Source: Sci Rep. 2021 Jan 26;11:2229. doi: 10.1038/s41598-021-82055-0 (PMC7838314; doi:10.1038/s41598-021-82055-0)

**Supplementary information**

**A PCR amplicon–based SARS-CoV-2 replicon for antiviral evaluation**

Tomohiro Kotaki^a*^, Xuping Xie^b^, Pei-Yong Shi^b^, Masanori Kameoka^a*^

^a^Department of Public Health, Kobe University Graduate School of Health Sciences, Japan

^b^Department of Biochemistry & Molecular Biology, University of Texas Medical Branch, Galveston, TX, USA

**Correspondence:**

Department of Public Health, Kobe University Graduate School of Health Sciences, 7-10-2 Tomogaoka, Suma-ku, Kobe, Hyogo 654-0142, Japan

Tomohiro Kotaki: [tkotaki@people.kobe-u.ac.jp](mailto:tkotaki@people.kobe-u.ac.jp)

Masanori Kameoka: [mkameoka@port.kobe-u.ac.jp](mailto:mkameoka@port.kobe-u.ac.jp)

**Supplementary Table S1.** Primer list for constructing a SARS-CoV-2 replicon with HiBiT-tag at the C-terminus of N protein.

| Name | Sequence | Description |
| --- | --- | --- |
| F1 Forward | 5’-AACGGTCTCATGATTAATACGACTCACTATAGATTAAAGGTTTATACCTTCCCAGGTAAC-3’ | For F1:  Encoding T7 promoter, 5’UTR, and ORF1a |
| F1 Reverse | 5’-AACGGTCTCAGCCGACAACATGAAGACAGTGTTTAGC-3’ |  |
| F2 Forward | 5’-AACGGTCTCACGGCCCAAATGTTAACAAAGGTG-3’ | For F2:  Encoding ORF1a |
| F2 Reverse | 5’-AACGGTCTCAGTTTGTAACACATCATACAAGTTGATG-3’ |  |
| F3 Forward | 5’-AACGGTCTCAAAACGTAATAGAGCAACAAGAGTCGAATG-3’ | For F3:  Encoding ORF1a |
| F3 Reverse | 5’-ACCGGTCTCATGTGAACATAACCATCCACTGAATATGTGC-3’ |  |
| F4 Forward | 5’-ACCGGTCTCACACACCTTTAGTACCTTTCTGGATAAC-3’ | For F4:  Encoding ORF1a |
| F4 Reverse | 5’-ACCGGTCTCAAAGGCATCTATGCTATTCTTGGGTGGG -3’ |  |
| F5 Forward | 5’-ACCGGTCTCACCTTCAAACTCAACATTAAATTGTTGGG -3’ | For F5:  Encoding ORF1a and 1b |
| F5 Reverse | 5’-ACCGGTCTCAACTCATAAAGTCTGTGTTGTAAATTGCGG -3’ |  |
| F6 Forward | 5’-ACCGGTCTCAGAGTGTCTCTATAGAAATAGAGATGTTGAC -3’ | For F6:  Encoding ORF1b |
| F6 Reverse | 5’-ACCGGTCTCATAAGTGTCTGAAGCAGTGGAAAAGCATG -3’ |  |
| F7 Forward | 5’-ACCGGTCTCACTTATGCCTGTTGGCATCATTCTATTGG -3’ | For F7:  Encoding ORF1b |
| F7 Reverse | 5’-ACCGGTCTCATCGTTTAGTTGTTAACAAGAACATCACTAG -3’ |  |
| F8A Forward | 5’-ACCGGTCTCAACGAACAAACTAAAATGTCTGATAATGGACCCC -3’ | For F8A:  Encoding N and a part of HiBiT-tag |
| F8A Reverse | 5’-TTAAGAAATCTTCTTGAACAGCCGCCAGCCGCTCACGGCCTGAGTTGAGTCAGCACTGC -3’ |  |
| F8B Forward | 5’-GGCTGTTCAAGAAGATTTCTTAAACTCATGCAGACCACACAAGGC-3’ | For F8A:  Encoding a part of HiBiT-tag, 3’UTR, and poly A |
| F8B Reverse | 5’-ACCGGTCTCATTTTTTTTTTTTTTTTTTTTTTTTTTTTTTTTTTGTCATTCTCCTAAGAAGC-3’ |  |

Underline: BsaI recognition site. Double underline: T7 promoter sequence. Wavy underline: HiBiT sequence. Dotted underline: poly A sequence.

*F8A and F8B fragments shall be merged by overlap PCR using F8A Forward and F8B Reverse primers to produce F8 fragment.

**Supplementary Table S2.** Primer sets for constructing a SARS-CoV-2 replicon with HiBiT-tag at the N-terminus of N protein*.

| Name | Sequence | Description |
| --- | --- | --- |
| F7 HiBiT-N Forward | 5’-ACCGGTCTCACTTATGCCTGTTGGCATCATTCTATTGG -3’ | For F7 HiBiT-N:  Encoding ORF1b and a part of HiBiT-tag |
| F7 HiBiT-N Reverse | 5’- ACCGGTCTCAAGCCGCTCACCATTTTAGTTTGTTCGTTTAGTTGTTAACAAGAACATCAC -3’ |  |
| F8 HiBiT-N Forward | 5’- ACCGGTCTCAGGCTGGCGGCTGTTCAAGAAGATTTCTGATAATGGACCCCAAAATCAGCG -3’ | For F8 HiBiT-N :  Encoding a part of HiBiT-tag and N |
| F8 HiBiT-N Reverse | 5’-ACCGGTCTCATTTTTTTTTTTTTTTTTTTTTTTTTTTTTTTTTTGTCATTCTCCTAAGAAGC-3’ |  |

Underline: BsaI recognition site. Wavy underline: HiBiT sequence. Dotted underline: poly A sequence.

*Primers for amplifying F1-F6 are identical to the sets shown in Table 1.

**Supplementary Table S3.** Primer and probe for qRT-PCR.

| Name | Sequence | Description |
| --- | --- | --- |
| CDC_2019-nCoV_N2-F | 5’-TTACAAACATTGGCCGCAAA-3’ | SARS-CoV-2 N gene detection |
| CDC_2019-nCoV_N2-R | 5’-GCGCGACATTCCGAAGAA-3’ |  |
| CDC_2019-nCoV_N2-P | 5’-[FAM]ACAATTTGCCCCCAGCGCTTCAG[BHQ1] -3’ |  |
| Hamster-GAPDH-F | 5’-GCACAGTCAAGGCCGAGAAT-3’ | GAPDH quantification for CHO-K1 and BHK-21 |
| Hamster-GAPDH-R | 5’-GCCTTCTCCATGGTGGTGAA -3’ |  |
| Human-GAPDH-F | 5’-GTCTCCTCTGACTTCAACAGCG-3’ | GAPDH quantification for 293T |
| Human-GAPDH-R | 5’-ACCACCCTGTTGCTGTAGCCAA-3’ |  |
| Hamster-ifn-b2-F | 5’-CTCCGCAAGAGACTTCCATC-3’ | IFN-β quantification for CHO-K1 |
| Hamster-ifn-b2-R | 5’-ACCAAACCTCCGACTTGTT-3’ |  |
| Hamster-mx1-F | 5’-CTTCAAGGAGCACCCACACT-3’ | Mx1 quantification for CHO-K1 |
| Hamster-mx1-R | 5’-CTTGCCCTCTGGTGACTCTC-3’ |  |

**
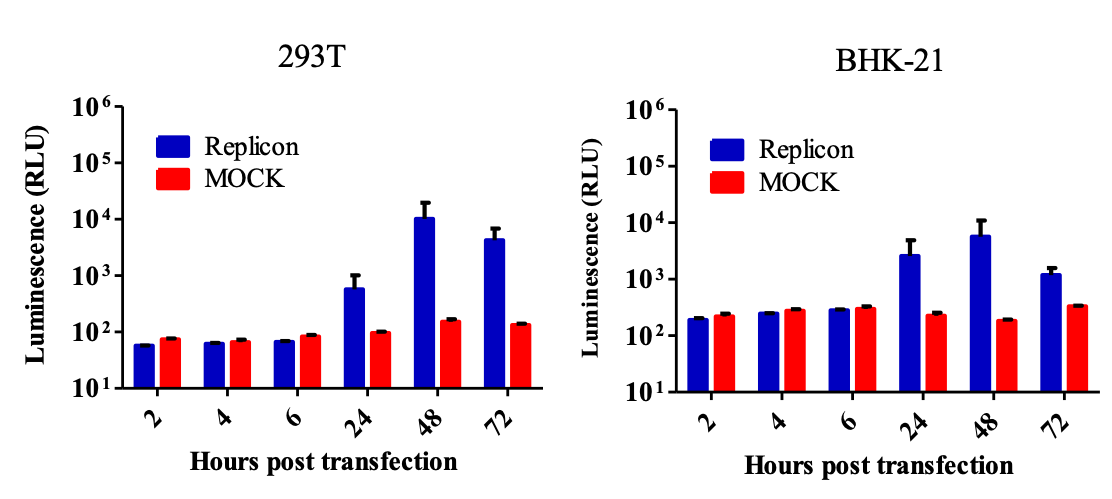
Supplementary Figure S1.** Characterization of a SARS-CoV-2 replicon in 293T and BHK-21 cells.

Kinetics of luminescence signals in 293T and BHK-21 cells. Cells were electroporated with 5 μg of replicon RNA. The intracellular luminescence signals were subsequently measured at the indicated time points. The mean and standard error of two independent experiments are shown in this figure.

**
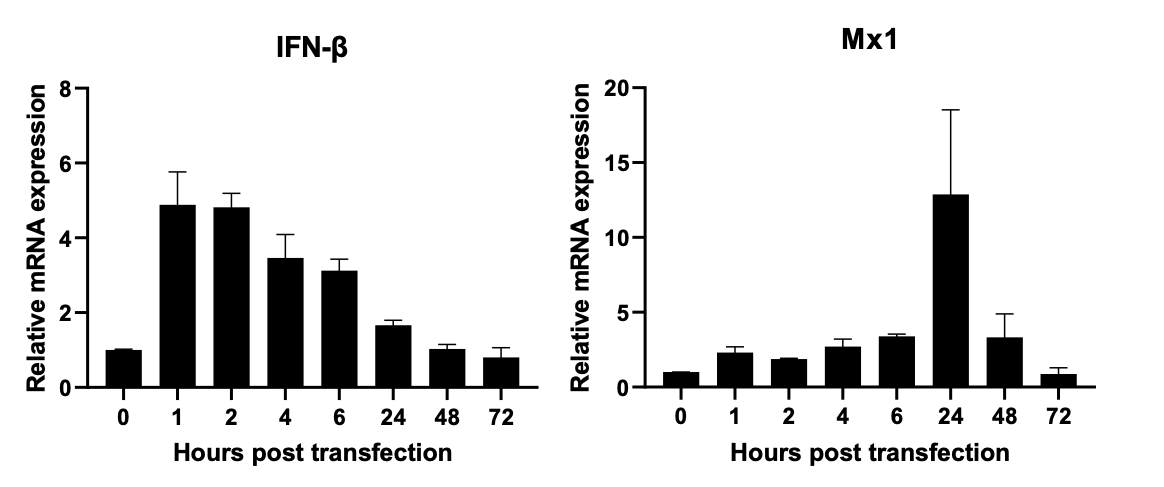
Supplementary Figure S2.** Quantification of the changes in IFN-β and Mx1 expression.

The CHO-K1 cells were electroporated with 10 μg of the replicon RNA. Total intracellular RNA was extracted from the transfected cells at the indicated time point. The relative mRNA expression of IFN-β and Mx1 was measured using qRT-PCR. The data was normalized to GAPDH expression. Results are presented relative to those of untransfected CHO-K1 cells (0 hpt). The mean and standard error of two independent experiments are shown in this figure.

**
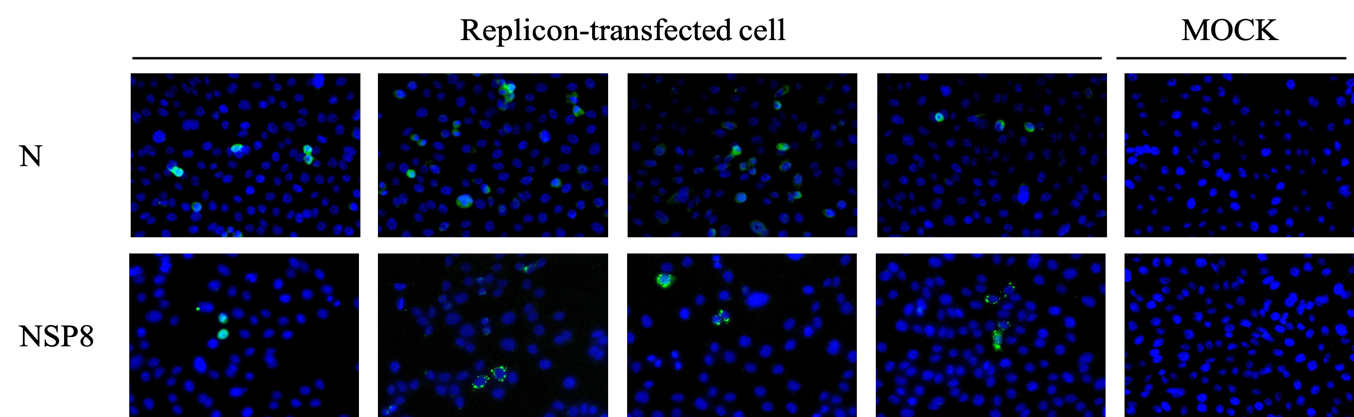
**

**Supplementary Figure S3.** Multiple IFA images for the detection of N and NSP8 proteins.

The CHO-K1 cell was electroporated with 5 μg of replicon RNA. The cells were fixed with 4% paraformaldehyde, followed by permeabilization with 0.5% Triton-X. The expression of N and NSP8 proteins were detected using anti-N or anti-NSP8 mAb, followed by incubation with goat-anti-mouse IgG conjugated with Alexa Fluor 488. Nucleus was stained by DAPI.

**
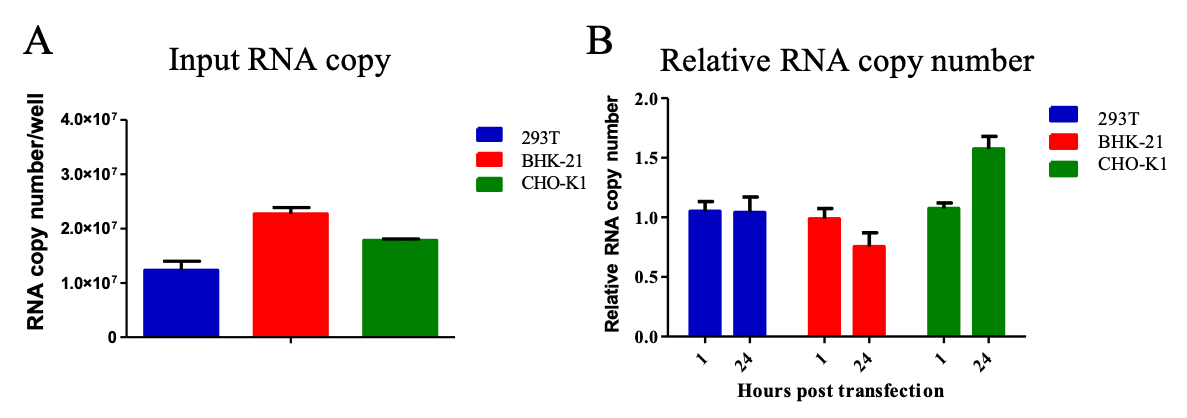
**

**Supplementary Figure S4.** Comparison of RNA copy numbers among the three cell lines.

1. Comparison of input RNA copy numbers among the three cell lines. Cells were electroporated with 10 μg of replicon RNA. At 1 hpt, total intracellular RNA was extracted from the transfected cells. Subsequently, replicon RNA copy numbers were measured using qRT-PCR. The mean and standard error of two independent experiments are shown in this figure.
2. Comparison of replicon RNA replication among the three cell lines at 24 hpt. Cells were electroporated with 10 μg of replicon RNA. The replicon RNA copy numbers were subsequently measured using qRT-PCR at the indicated time points. The result was later expressed as relative RNA copy number compared to that at 1 hpt. The mean and standard error of two independent experiments are shown in this figure.

**
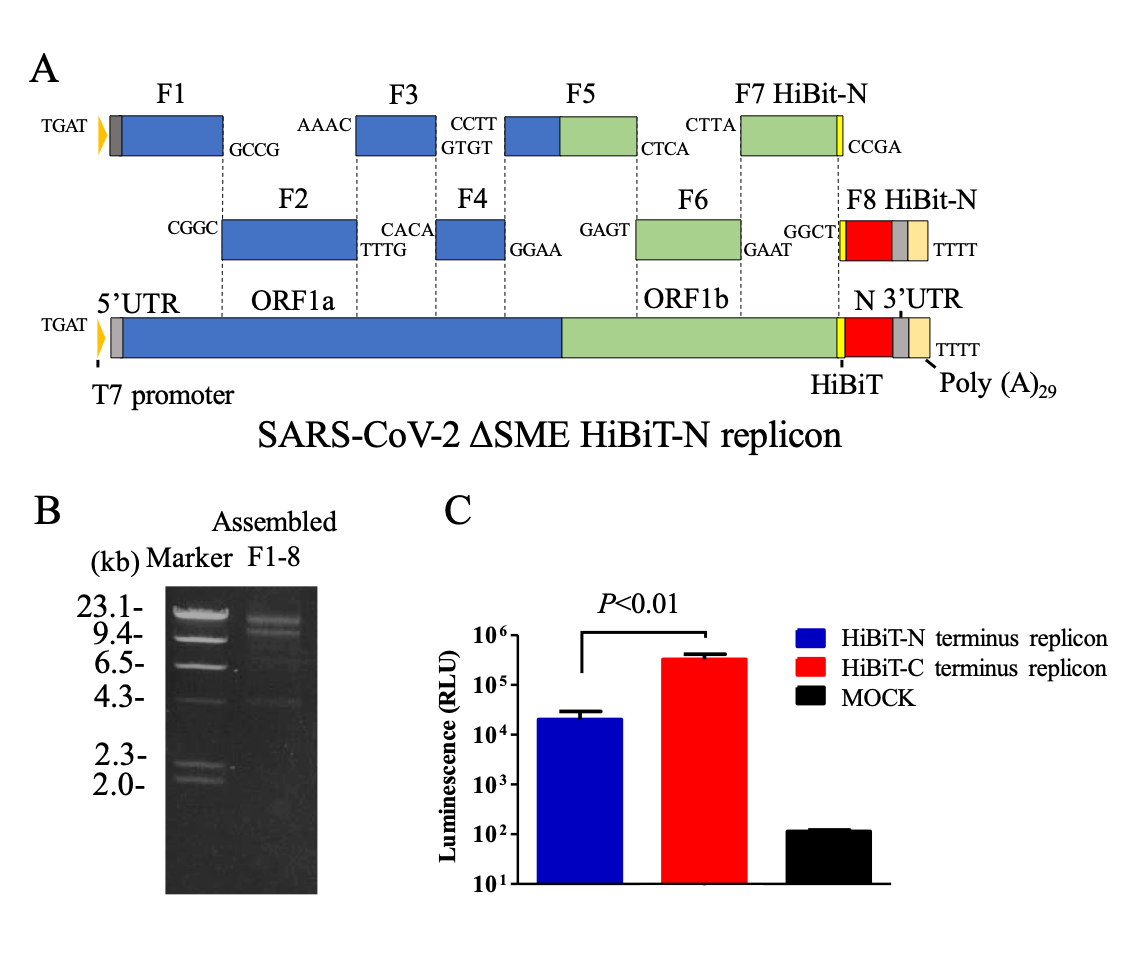
Supplementary Figure S5.** Construction and characterization of a SARS-CoV-2 replicon with HiBiT-tag at the N-terminus of N protein.

1. Strategy for *in vitro* assembly of a SARS-CoV-2 replicon DNA with HiBiT-tag at the N-terminus of N protein. The nucleotide sequences of the overhang are indicated in this figure. The replicon DNA was assembled using *in vitro* ligation.
2. Electrophoresis of an assembled DNA. About 100 ng of assembled DNA was run on a 1% agarose gel. The λ-HindIII digest marker is indicated in this figure. Successfully assembled replicon DNA was 23.2 kb. Original unedited gel image is shown in the supplementary dataset and image was not joined from different parts of the gel.
3. Luminescence signals at 24 hpt. CHO-K1 cell was electroporated with 5 μg of replicon RNAs. Intracellular luminescence signals were measured at 24 hpt. The mean and standard error of two independent experiments are shown in this figure. A t test was performed to determine the statistical significance.

**Original gel images**

Framed area indicated the cropped image as seen in the Figure 1 and Figure S1. No figures were cropped and joined together from different images. Contrast adjustment was applied equally across the entire image.


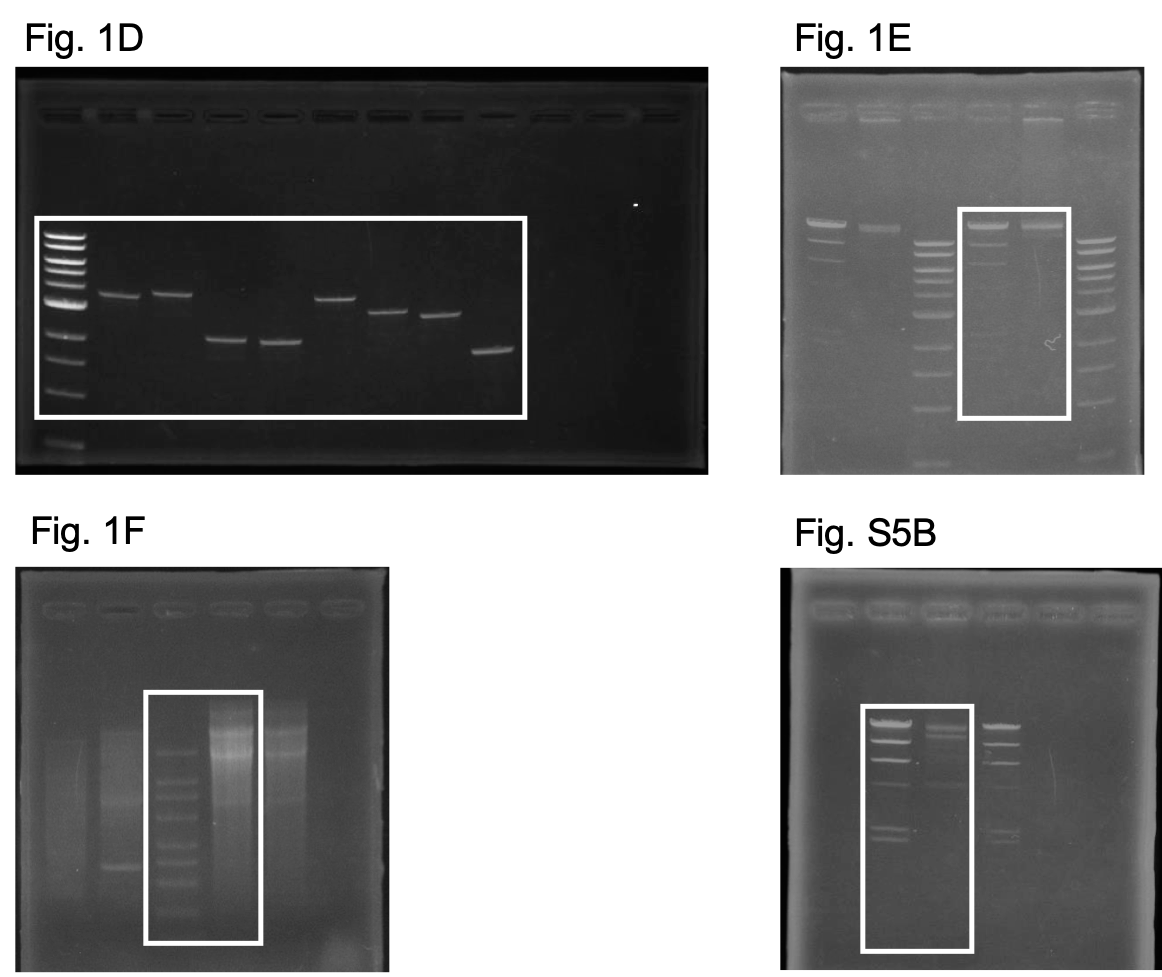

Supplement: Supplementary file 1 — Supplementary Information [file 41598_2021_82055_MOESM1_ESM.docx]
